# Abdominal Surgery in the Private Residence

**Published:** 1903-07

**Authors:** Marcus F. Carson

**Affiliations:** Griffin, Ga.


### ABDOMINAL SURGERY IN THE PRIVATE RESIDENCE.*

BY MARCUS F. CARSON, M.D.,

GRIFFIN, GA.

My purpose in selecting this subject for my paper is not to discuss the disadvantages of the sanitariums (for in my opinion they have none), but to bring before the profession what I believe to be a very much exaggerated disadvantage of operating in the private home.

A few years ago before a sanitarium was so essential to a reputation, it was no uncommon thing to find our best surgeons living in the smaller towns and doing good work in the private home.

To-day the trend of public opinion is towards the sanitariums. The question naturally arises with physicians living in small towns, is it because that it is so much better for the patient that he be operated on in a sanitarium, or is it that it benefits some one else?

If it be true, as the public has been taught, that good surgery can be done and good results obtained only in sanitariums, then those of us who have not access to these institutions must either establish a sanitarium, or abandon that most profitable branch of our profession, and advise our patients to go to sanitariums. If it is not true, and we can prove it by practical results, then it is our duty and to our interest to present our side of this question and endeavor, if possible, to overcome the public sentiment against operating in the private house.

What are some of the disadvantages of operating in the private residence? First, asepis, by far the most essential thing to successful surgery. I am confident that by the exercise of the proper precaution, that by the same method and for the same expense, we can make the room in the private home just as fit for surgical work as it is possible to make a room in any sanitarium.

If the surgeon who operates in the house sees to it that all furniture and curtains are removed, the floor thoroughly rubbed with a strong solution of bichloride of mercury, that every article and instrument that comes in contact with the patient is thoroughly

* Read at Columbus meeting Georgia Medical Association sterilized; in other words, if we use the same methods and exercise the same precaution in the private home that we would in the sanitarium, we will get the same results. If this be possible, then we have overcome the first and main objection to the private room.

Second, the question of trained nurses. The laity exhibits a blind confidence in the trained nurse that is marvelous. I have always found that the best nurses are those who carry out the surgeon's instructions best, regardless of costume. I have never had any difficulty in securing a nurse with sufficient intelligence to do this. When the surgeon can see the patient two or three times each day and attend to all the dressings himself, it is not so necessary that the nurse be so highly educated or so well trained. After all, it is good results that are to be desired.

In order to learn what the mortality really is in operating under such grave surroundings, I took the trouble to address letters to fifteen physicians doing general work in towns where there are no sanitariums. I received replies from twelve of them, who reported sixty-five abdominal sections with one death. They were as folfows; forty-four operations for appendicitis, one death; sixteen ovariotomies, no death; five hysterectomies, no death.

These were not selected cases, such as could go away for operation, but many of them emergency cases. They were not all operated on by the same surgeon, but nearly equally divided among several.

I have not been able to secure statistics from any of our sanitariums, but if they can show a smaller death-rate I would be glad to hear from them.

I know it is difficult to compare results in this particular line of work unless we had a clear record of each individual case. Neither is it a fair test of the surgeon's skill, but it does show that aseptic surgery is not only possible but eminently practicable in the private residence.

It is true that it is easier on the surgeon to have all of his cases at the same place, and to have some one else to attend to details, but I for one am willing to do all these myself for the fee charged by the sanitarium.

But these splendid equipments and conveniences do not effect results. I claim no advantage for the country residence over the sanitarium, not even that of pure atmosphere, for the old practice of physicians living in cities seems to have been reversed, and instead of advising their patients to go to the country for pure oxygen, they are advised to go to the cities. I have noticed in the advertisment of our best sanitariums every room has a sunny exposure and the ventilation is perfect.

A third argument against the surgeon living in the smaller towns is that only a specialist should undertake such work, and that, by reason of much experience, he is better qualified for such work. If the general practitioner advises his patient to consult a specialist for every disease there is a specialist for, he will soon realize that there will be little left for him to do. I know that it is to the specialist's interest for the people to believe that work in his particular line can be done better by one doing that work exclusively. It is also to his interest for the public to believe that it is very dangerous for the general practitioner to undertake so simple an operation as the cauterization of the schneiderian membrane, to say nothing of that very common malady, with which almost everybody seems to be afflicted, bones in the nose.

If it is dangerous for the general practitioner to do this work, it is wrong for the specialist to say by his signature to a diploma that he is qualified to do it.

I desire to report briefly six of my own cases, not because they are specially interesting, but because they were operated on under so-called dangerous surroundings.

My first case was a woman, aged seventeen. Diagnosis, ovarian cyst. On January 10, 1902, I operated on this case, and removed an ovarian cyst which contained nearly five gallons of fluid. Patient made a rapid recovery. I operated on this case in a cabin with four rooms. I selected the best ventilated room, and for the sum of five dollars and a little work I made it clean. I saw this case twice a day, attended to all the dressing myself, and allowed nothing to come in contact with patient that was not sterilized.

Case number 2 was a young lady, aged seventeen. Diagnosis, appendicitis. On January 12, 1902, I operated, removing the diseased appendix with several ounces of pus.

In this case I used gauze as drainage. On the fourth day I removed gauze, closed wound, and patient made a complete and rapid recovery. This case was operated on in a farmhouse made clean under my own supervision at a very small expense.

Case number 3, diagnosis, cystic ovary. On August 14 I operated on this case in a room in a private residence that I disinfected myself.

I removed an enlarged cystic ovary, and patient made an uninterrupted recovery. I attended to all the dressings myself, and where we can do this there is no need of a trained nurse.

Case number 4 was a young man, aged twenty-two. I saw patient on September 1, 1902. Diagnosis, appendicitis, and on the same day removed a diseased appendix, closed wound, and patient recovered without a stitch abscess.

My fifth case is not a laparotomy, but it presents some points of interest which I would like to hear discussed, and was done in the private home.

On March 20 I was called to see this case with another doctor. Patient, male, eleven years of age, five weeks ago had a severe attack of pneumonia. On examination I found the heart very much displaced, with the apex to the right of the median line, complete dullness from the eighth rib to the clavicle, temperature 102½, pulse 120. I diagnosed the case empyema, and suggested an operation.

On March 21 I did the following operation. I made an incision between the sixth and seventh ribs in the axillary line, opening the pleural cavity. I then introduced a flexible metallic probe with an eye in its point, made it project between the eighth and ninth ribs, and then cut down on the probe, and passed it out the lower wound. I then attached to it a large drainage-tube, and carried it through the pleural cavity. From this patient I drew off forty ounces of pus, and, judging from the dressings shortly afterwards, don't believe I took more than half of it. I feel sure that this case was a result of pneumonia.

In acute pleurisy with effusion we have points of very great interest. We may divide them into cases of acute pleurisy coming on idiopathically from a chill, and cases coming on in the course of acute fevers. I once heard Mr. Treves say that the majority of caught-cold cases were tubercular. The after-history of these cases shows that if the original disease was not tubercular tubercle became an element in the later history. If this be true, we should be very careful in our prognosis in cases of acute pleurisy coming on without any other cause than a chill.

The main points in treating these cases are when to draw off the liquid and how much to remove. We used to be taught two things in connection with this. First, that we should draw off the fluid until the temperature is down. Second, the fluid should never be drawn off in tubercular cases. But according to modern ideas both these injunctions are incorrect.

I believe we should draw off the fluid if it is embarrassing, whatever the cause or circumstances, notwithstanding we are taught not to draw it off where there is tuberculosis of the lungs. Second, how much to draw off. I believe that we should be guided by the pulse and presence of cough. At the first cough I believe we should withdraw the needle. If the quantity of fluid is sufficient to displace the heart, the removal of the entire quantity at one time, allowing the heart to suddenly resume its normal position, together with the removal of the pressure of the liquid, is likely to cause a collapse.

The main advantage in establishing two openings, as I did in this case, is that a drainage-tube is passed through the pleural cavity in its lower portion, and gives perfect drainage, and even in the worst cases of empyema such drainage will result in a cure without the usual fistula, which often necessitates Estlander's operation. This case made a complete recovery.

Case 6 was an external urethrotomy without a guide. Patient, a man, aged thirty, ten years ago had gonorrhea, and has had trouble in passing his urine ever since.

On March 27 I was called and found him unable to pass his urine, very weak and in a great deal of pain. I made an effort to enter his bladder through the urethra, but was unable to pass the smallest bougie. I suggested an operation, and, on March 28, I anesthetized patient and made a median perineal section without a guide. I entered the urethra just behind the stricture. I then introduced a large size rubber tube into the bladder and drew one half gallon of urine. Then with an Otis urethrotome I did an internal urethrotomy, and enlarged the urethra sufficiently to pass a number 32 sound. This case was operated on in his bedroom and got well.

